# Diversity of culturable thermophilic bacteria from Tata Pani hotspring of Kotli Azad Jammu and Kashmir

**DOI:** 10.3897/BDJ.11.e99224

**Published:** 2023-03-14

**Authors:** Kazima Ishaq, Asad Hussain Shah, Anila Fariq, Sajida Rasheed, Sammyia Jannat

**Affiliations:** 1 Department of Biotechnology, Faculty of Basic and Applied Sciences, University of Kotli Azad Jammu and Kashmir, Kotli, Pakistan Department of Biotechnology, Faculty of Basic and Applied Sciences, University of Kotli Azad Jammu and Kashmir Kotli Pakistan; 2 Senior Research Fellow, Faculty of Biology Medicine and Health The University of Manchester The Michael Smith Building Oxford Road Manchester M13 9PT, Manchester, United Kingdom Senior Research Fellow, Faculty of Biology Medicine and Health The University of Manchester The Michael Smith Building Oxford Road Manchester M13 9PT Manchester United Kingdom

**Keywords:** Hot water springs, thermophiles, diversity, bacteria, archaea, 16S rDNA

## Abstract

Hot water springs are unique areas populated by mesophiles, thermotolerant and hyperthermophiles. They are the source of diversity of thermophiles, mainly belonging to archaea and bacteria domains. The diversity of thermophiles gives an outline of the huge biological potential that can be exploited for industrial applications.To this end, this study was aimed to isolate and characterise the unexplored thermophilic microorganisms from hot water spring in Tatapani, Tehsil & District Kotli AJK, Pakistan. Around 10 bacterial isolates were identified using morphological, biochemical, physiological and molecular attributes. Sequencing of the 16S rDNA gene of the isolates followed by BLAST search revealed that the strain MBT008 has 100% similarity with *Anoxybacilluskamchatkensis*. MBT012 showed 99.57% similarity with *A.mongoliensis*, MBT014 was affiliated with *A.tengchongensis* with 99.43% similarity, MBT009 showed 99.83% homology with *A.gonensis* and MBT018, 98.70% similarity with *A.karvacharensis*. The presence of all this microbial diversity in one common source is of immense importance related to envioronmental and industrial aspects in general and extraction of thermostable enzymes from these thermophiles specifically opens new horizons in the field of industrial biotechnology. These thermophiles are revealing new capabilities and are being manipulated by biotechnologists in utilizing them in different unique ways.

## Materials and methods

**Sample collection**

A total of ten water/sediment samples from 10 different locations separated by a distance of approximately 1.0 ft (0.3 m) were collected from different locations of the hot water spring of Tatapani (District Kotli) in sterilised screw-capped bottles and were carried to the laboratory of Biotechnology and processed immediately. The physiochemical characteristics of the hot water spring was examined. The electrical conductivity was monitored by using an EC meter and pH of water samples was measured using a pH meter.

**Isolation of thermophilic strains**

The sediment and water samples were incubated at 70°C overnight in nutrient broth medium for the isolation of thermophiles. The growth was observed by analysing the turbidity of the growth medium. Next day, the suspended cultures were poured by spread plate technique on the agar plates for the isolation of the mixed population of microorganisms. For calculating the colonyforming unit of viable bacterial cells, several serial dilutions were made in 1:10 ratios. From these dilutions, 0.1 ml was plated on to nutrient agar and, after 24 hours incubation, colonies were observed and counted on the plate. The number of bacteria (CFU) per millilitre of sample was calculated by dividing the number of colonies by the dilution factor. To purify bacterial colonies, single colony streaking is done by picking individual colonies with a sterilised inoculation loop and touching it iton to the nutrient agar plate.

**Morphological characterisation**

Colony morphology like colour, shape, elevation and texture of pure bacterial cultures obtained through single colony streaking was observed visually as well as examined under the microscope. Gram’s staining was performed to differentiate bacteria according to cell wall composition through a series of staining and decolourisng steps.

**Growth conditions optimisation**

Growth conditions were optimised for pH, temperature, inoculum size and incubation time. Temperature range for incubation varied from 40, 50, 60, 70 and 80°C and the pH dependence of growth was tested in the pH range of 6.0-9.0 in nutrient broth medium, while the effect of varied incubation time intervals for these thermophiles’ growth was noted for 24 hours, 48 hours and 72 hours. The growth was also monitored at different inoculum size i.e. 25 µl, 50 µl, 75 µl and 100 µl. The optical density in all conditions was measured to measure the growth of strains at 600 nm on a double beam UV/VIS scanning spectrophotometer (Model: AE-S90-MD).

**Biochemical characterisation**

For biochemical characterisation, API 20E strips were used to examine the isolates for numerous substrates utilisation like adonitol, glycerol, D-arabinose, erythritol, L-arabinose, D-xylose, L-xylose, ribose, Β-methyl xyloside, mannose, glucose, L-sorbose, dulcitol, rhamnose, inositol, mannitol, sorbitol, D-methyl-D-glucoside, amygdalin, N-acetylglucosamine, aesculin, arbutin, salicin, maltose, cellobiose, lactose, trehalose, sucrose, melibiose, raffinose, starch, glycogen, D-turanose, D-fucose, inulin, L-fucose, D-arabitol, D-lyxose, L-arabitol, gluconate, 2,5-ketogluconate and xylitol. Suspended bacterial cultures (100 µl) grown for 24 hours in 0.8% sterilised saline solution were injected into the wells of strips containing the substrate and incubated at 70°C for 24 hours. The colour change was studied according to kit instructions. After the incubation period, all the results were recorded and then revealed the tests which require the addition of reagents. The presence of oxidase and catalase enzymes was examined according to the procedures designed by Prescott et al. (1997).

**Antibiotic Sensitivity**

The sensitivity profiles of the bacteria isolated from water/sediment samples against various antibiotics like ciprofloxacin, levofloxacin, azithromycin, cefixime, linezolid etc. was also done by the disc diffusion method.

**DNA extraction and amplification of the 16S rDNA gene**

Genomic DNA was extracted from bacteria consistent with the protocol described by Cheng and Jiang (2006). The 16S rDNA gene amplification from the purified genomic DNA was performed in 0.2 ml PCR tubes with 20 μl reaction volume using the forward primer 27F AGAGTTTGATCCTGGCTCAG and the reverse primers 1492R TACGGCTACCTTGTTACGACTT and all the PCR amplifications were carried out by using a thermal cycler (Model: TC-TE).

**Sequencing analysis**

After amplification, the PCR products obtained were sequenced by using same upstream and downstream primers, by a commercial sequencing facility i.e. MacroGen. The entire 16S rDNA sequences of these bacterial strains obtained were blasted using the online NCBI BLAST programme (Waterhouse et al. 2009). Phylogenetic analyses were done to express evolutionary associations of these isolates. The analysis started with aligning of sequences by the Clustal W tool and after alignment, the phylogenetic tree was built using MEGAX software.

## Introduction

Hot springs comprise of the environment with high temperature ranges and formed due to geothermal movement and are host to variety of thermophiles (Zhang et al. 2004, Yohandini et al. 2015). A hydrothermal spring is characterised by warm groundwater which is heated by intense geothermal heat, the indispensable heat beneath the Earth’s surface. The life in hot springs started long before reaching the surface and the hot water serves as a habitat for large number of bacteria and algae which were then termed as thermophiles (Adiguzel et al. 2009). These thermophilic microorganisms prefer to grow at high temperature which normally does not exist in nature. Thermophilic microorganisms were the first living organisms which develop and evolve in the ancient days when the Earth’s surface temperature was relatively high, so they were called the “Universal Ancestor” (Kumar 2010). Moreover, a study of thermophiles resulted in the discovery of novel species (Kozina et al. 2010).

Thermophiles are characterised into moderate (optimum temperature, 55–60°C), extremophiles (optimum temperature, 60–80°C) and hyperthermophiles (optimum temperature, 80–110°C) (Saha et al. 2005). Nowadays, extremophilic microorganisms with their different classes, thermophiles, alkaliphiles, acidophiles, halophiles and psychrophiles, attracted the attention of many researchers in various areas, because of their capability to resist and perform life functions under extreme environmental conditions (Tango and Islam 2010). Due to their potential to produce thermostable enzymes (like DNA polymerases, chitinases, cellulases, amylases, pectinases, proteases, lipases and xylanases), thermophilic bacteria have attained great consideration amongst extremophiles. These thermostable enzymes are more stable and possess unique characteristics that make them appropriate for performing biotechnological procedures at higher temperatures (Singh et al. 2011). The earliest thermophilic spores forming bacteria capable of surviving at 70°C were discovered by Miquel in 1888 for the first time (Miquel 1888). Since that, numerous spore forming strains of thermophilic bacteria, mainly related to *Bacillus* and *Clostridium* genera, have been characterised (Maugeri et al. 2001, Belduz et al. 2003).

Thermophiles have attracted the attention of many scientists for their potential in biotechnological processes (Adiguzel et al. 2009). These thermophilic bacteria have been characterised phenotypically and genotypically in particular from many hot springs in different regions of the world, including Turkey (Gul-Guven et al. 2008), Bulgaria (Derekova et al. 2008), Italy (Maugeri et al. 2001), China (Poli et al. 2009), Greece (Sievert et al. 2000, Lau et al. 2008), Iceland (Takacs et al. 2001) and India (Sharma et al. 2008). Due to development in techniques of molecular biology, such as gene sequencing of 16S rRNA, tremendous opportunities have become available for identifying and characterising microbes at species level.

In recent times, the bio-chemical and molecular characterisation of thermophilic microorganisms from geothermal springs has been reported to explain the mechanism and molecular cause of the adaptation of these organisms to extreme temperature (Panda et al. 2016).

**Figure 1.**
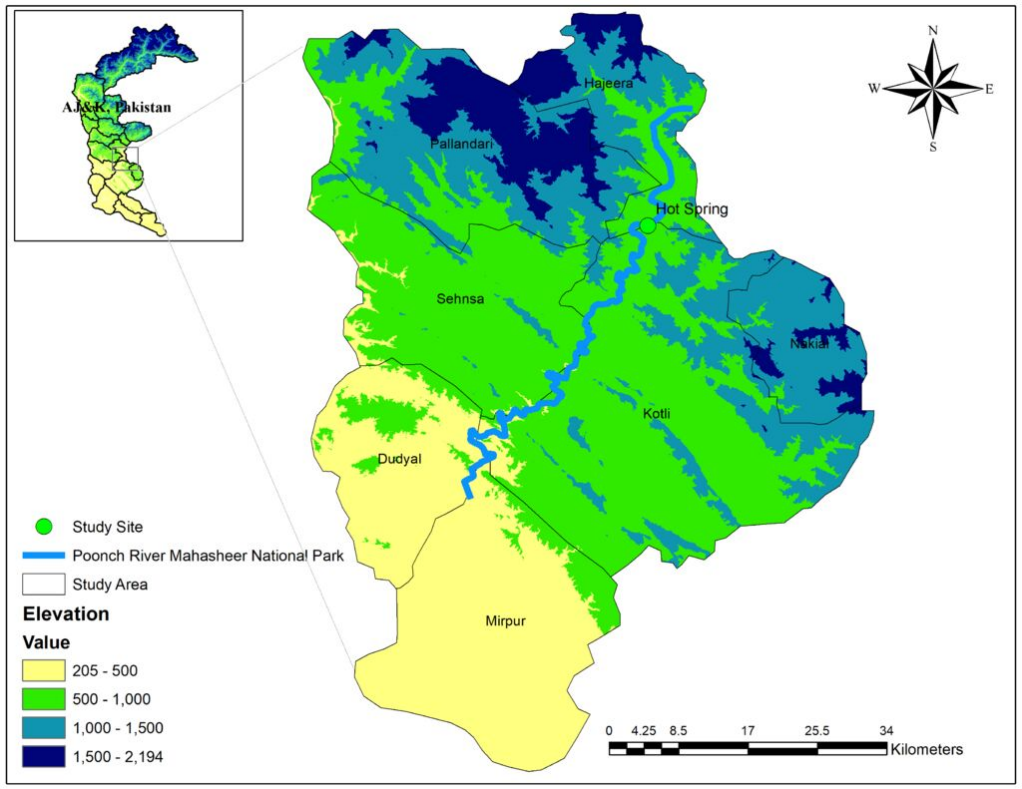
Map of the hot spring Tatapani, AJK, Pakistan.

Tatapani is situated in Kotli, Azad Kashmir (Pakistan) at a latitude and longitude of 33.60 N, 73.94 E, respectively and TattaPani hot springs are located on the right bank of Poonch River as shown in Fig. 1 and can be reached from Kotli by covering a distance of 26 km. The hot spring of Tatapani has been known for its flowing hot water on the western bank of Poonch River at an elevation of 682 m (2,237 ft). Due to its exclusive hot water, it attracts the attention of tourists who benefit from its steam in winter. Locals and tourists enjoy this hot water bath which is rich in sulphur. The water is believed to have high medical value and good for the skin. However, the microbial diversity of this very unique repository of microorganisms has not been explored. The tremendous capability of such important bacterial strains can be a substantial source of thermostable enzymes.

Tatapani hot spring has not yet been explored from the microbiological characteristics. The specific purpose of this study was to identify and characterise the bacterial strains from the hot water spring of Tatapani by using various morphological markers and to carry out the biochemical and molecular characterisation of the isolated bacteria. Due to limited scientific knowledge existing on the research being done in this field and to explore this unseen and under-utilised source of thermostable bacteria, this study was performed to isolate and characterise the thermophilic microorganisms from various locations of the hotwater spring situated in Tatapani, District Kotli AJK, Pakistan. The advancement of the scientific information regarding the biodiversity of these delicate ecosystems underlines the requirement for their preservation; in addition, this microbial diversity possibly will be the basis for further biotechnological utilizations representing an important keystone for a developing region.

## Results

**Isolation and Characterisation of the Samples**

In this study, the hot spring represented a moderate to high temperature (39.9–75°C) and neutrophilic to alkalophilic (pH 7.03–8.6) environment with varying electrical conductivity (0.51– 3.27 µscm^_−1_^). After enrichment, visible turbidity was observed in almost all samples within 24 to 48 hours, frequently appearing clumpy or as a surface pellicle. After the turbidity had become equally thicker, the colour of some of the tubes containing the microbial sample was yellow or orange. The total cell count varied from one sampling site to another (Table 1). The enrichment samples were given codes viz. MBT006, MBT007, MBT008, MBT009. MBT010, MBT011, MBT012, MBT013, MBT014 and MBT018.

The variation of colonies count was observed at different sampling sites. These ten samples were cultured at 70°C. It was noted that the number of CFUs in sample 1 i.e. MBT006 is greater (6.0×10^3^) and decreased as we move away from the main hot water source towards sites 2, 3, 4 and so on.

**Identification and differentiation of isolates**

The bacterial strains exhibited different colony and cell morphology. Under light microscope, all were detected as rods either single or were arranged in chains. Within 24 hours of incubation at 70°C, compact spreading colonies were seen which were yellow, white, creamy, brown and greyish white in colour.

Morphologically, the strains exhibited some difference in the colour, shape, texture and margin of the colonies (Table 2). They appeared creamy, white and grey; translucent or opaque; smooth or rough; with regular or irregular edges. The colonies appeared either raised or flat on the agar surface.

**Biochemical and Metabolic Characterisation of the bacterial Isolates**

The biochemical characterisation was done by API 20E strips for identifying phenotypic diversity and none of the ten isolates shared the common phenotypic characters (Table 3). All the isolates were positive for β-galactosidase and citrate utilization. Most of the isolates were unable to metabolise many sugars i.e.nositol, mannitol, sorbitol, rhamnose, melibiose, sucrose and amygdalin. However, a few isolates showed glucose and arabinose utilization (Table 4). Only two isolates i.e. MBT011 and MBT012 exhibited positive results for indole production. All except two (MBT006 and MBT012) were able to produce gelatinase enzyme.

**Antibiotic sensitivity**

The antibiotic sensitivity profile of the bacterial isolates showed that all isolates were sensitive to amoxicillin (Ax) and levofloxacin (LEV) and resistant towards metronidazole (MET). The strains MBT009 and MBT012 were found resistant to clarithromycin (CLR) and linezolid (LNZ), while most of the strains showed resistance against AZM (Table 5).

**Physiological characteristics**

**Figure 2.**
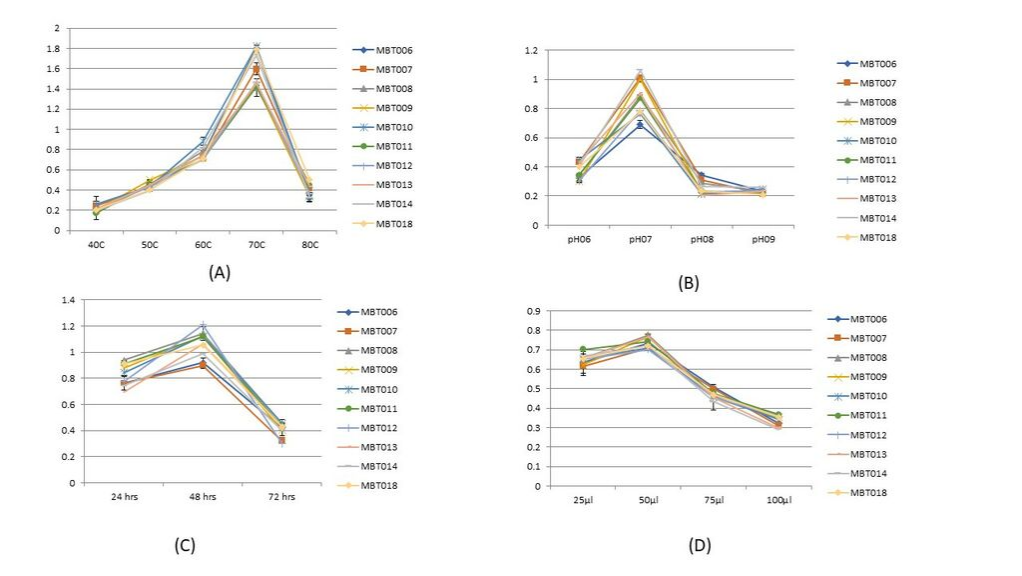
Measuring growth rate of bacteria. (**A**) Growth rate of isolates at different temperatures. (**B**) Growth rate of isolates at different pH; (**C**) Growth rate of isolates at different incubation periods; (**D**) Growth rate of isolates at different inoculum size.

Physiological studies were performed with all the ten strains. Temperature (a physiological limiting factor) controls the microbial cells multiplication. The temperature of water governs distribution of microbes within hot springs. The association between growth rate and temperature is shown in Fig. 2. The optimum temperature for their growth was 70°C, the maximum growth temperature was 80°C and the minimum temperature was about 40°C. The maximum growth of bacterial isolates was observed when incubated overnight at 70°C. At 40°C and 80°C, no growth was observed; minimum growth was appeared after incubation at 50°C and 60°C. These results depict that the optimal temperature for growth of these isolates was 70°C.

In addition to temperature, pH of water is an important factor defining the microbial diversity in hot water springs. All isolated strains were inoculated in LB broth with different pH ranges from 6.0-9.0. Good growth was noted at pH 7 and it is considered as the optimum pH for the growth of thermophilic bacteria as shown in Fig. 2.

The growth rate was also measured at varying time intervals in order to obtain the optimum growth period. Best growth was observed after 48 hours of incubation. From these results, the optimum time for growth of thermophiles was considered at 48 hours. Similarly, the growth rate of thermophilic bacteria was observed best when incubatd with 50 µl of inoculum.

**Molecular characterisation of the Isolates**

For the ultimate identification and phylogenetic study of the strains, 16S rDNA gene sequencing was done following the Basic Local Alignment Search Tool (BLAST) programme. The 16S rDNA gene sequences of the five isolates were aligned with their associated bacterial sequences from the GenBank databases. Sequence analysis revealed high affiliation with those of the linked strains available in GenBank. The sequence alignment showed that all the strains belonged to genus *Anoxybacillus* with significant sequence similarity as listed in Table 6.

**Figure 3.**
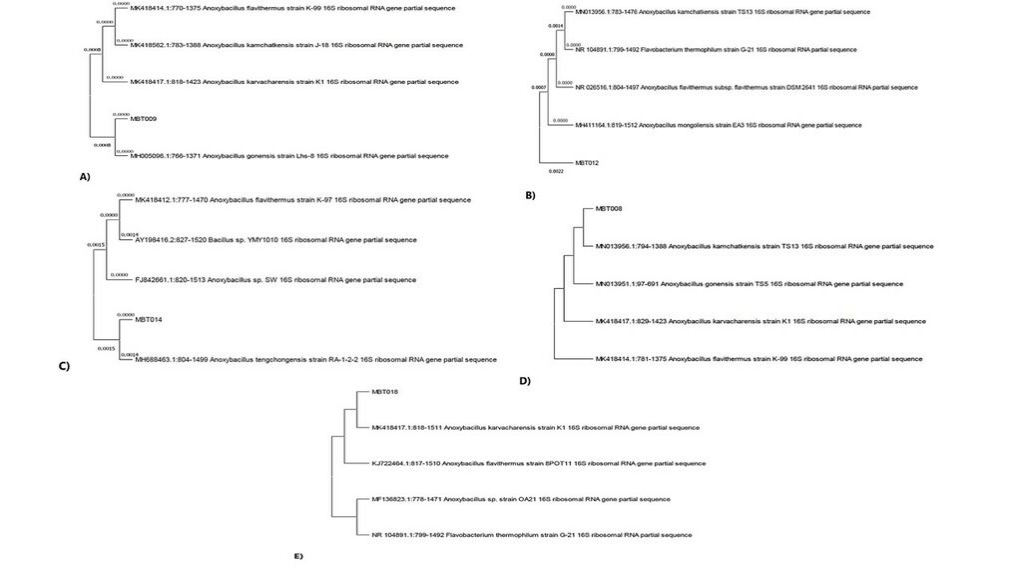
Phylogenetic trees of Tatapani hot spring isolates which have the highest homology with the genus *Anoxybacillus*. The phylogenetic trees were constructed using the neighbour-joining method, based on total 16S rDNA sequencing with 100 bootstrap replicates (software MEGAX). **(A)** MBT009 showing similarity with *A.gonensis*; **(B)** MBT012 showing similarity with *A.mongoliensisas*; **(C)** MBT014 with *A.tengchongensis*; **(D)** MBT008 with *A.kamchatkensis*; **(E)** MBT018 was closely related to *A.karvacharensis*.

The phylogenetic tree was constructed for all the isolated strains, based upon 16S rDNA gene sequence alignment. BLAST analysis showed the strongest similarity (100%) of the strain MBT008 with *A.kamchatkensis* and MBT009 (99.83%) with *A.gonensis*. MBT012 showed 99.57% similarity with *A.mongoliensisas*, MBT014 with *A.tengchongensis* (99.43%) and MBT018 was closely related to *A.karvacharensis* (98.70%) (Fig. 3).

The genus *Anoxybacillus* was abundant in the majority of the explored locations; the presence of *Anoxybacillus* in the majority of sampled sites is ascribed to the capability of the genus to pass at higher rates, as well as their potential to resist extreme environmental stresses.

## Discussion

Geothermal environments are enriched by the diversity of thermophilic archaea and bacteria. Many thermophilic and hyperthermophilic archaea have been isolated from geothermal and hydrothermal systems. In order to obtain a better understanding of microbial ecosystems and roles in the geothermic community, many studies have been performed to reveal the link between microbial niches, diversity and physicochemical factors, such as temperature, pH and water chemistry (Skirnisdottir et al. 2000, Purcell et al. 2007, Pearson et al. 2008, Everroad et al. 2012, Wang et al. 2013). The hot spring of Tattapani is a habitat for the diversity of thermophilic microorganisms and it represents the functional adaptations of these microbes to withstand extreme temperatures.

One of the studies (Nakagawa and Fukui 2002) reported that the bacterial communities in streamers with a temperature above 65°C was abundant in Thermus, Thermodesulfo bacteria, Crenarchaeota and Aquificae. In 1995, Miquel firstly characterised the aerobic thermophilic, spore forming bacteria, which were able to grow at 70°C (Miquel 1995).

Bacteria belonging to the genus *Thermus* had become important in thermophile research after the isolation of *Thermusaquaticus* carried out by Brock and Freeze (1969). Later on, numerous additional *Thermus* species have been discovered. However, in this study, the existence of thermophilic bacteria related to genus *Anoxybacillus* were investigated in thermal spring of Tatapani, Pakistan. All of the isolated thermophiles belong to the domain bacteria, phylum firmicutes, class bacilli, order bacillales, within family bacillaceae (http://rdp.cme.msu.edu/ seqmatch). Therefore, it is clear that the water sample enriched with NB media was appropriate for growth of the genus *Bacillus*.

The decrease in CFU with change in altitude is attributed to the gradual decrease in temperature of water when the sample was taken some distance away from the hot spring. The temperature significantly influenced the bacterial richness. Occurence of bacteria, at elevated temperatures, is due to numerous adaptations in physiological conditions and genetics as the stress response to maintain homeostasis (Wang et al. 2015).

Microorganisms grow across a wide temperature range, pH, salinity and oxygen levels. Extremophilic microorganisms have the potential to grow in diverse extreme environments, such as low or high temperature, alkaline and acidic pH, high radiations and high salinity (Mirete et al. 2016).

The optimal temperature observed for maximum growth of isolates in our study was 70ºC, optimal pH observed was 7.0 and optimal time interval was 48 hours. Large-scale comparisons of genomes of mesophiles and thermophiles had confirmed that genomes of the thermophiles contain a higher guanine and cytosine (GC) content than that of mesophiles (Takami et al. 2004, Wang et al. 2015). It has been established that a high GC content is related to the thermal stability of the genome and optimal growth temperature of microbes (Musto et al. 2005, Musto et al. 2006). In addition, rRNAs and tRNAs, that serve as the translational machinery for most of the thermophiles, were reported to contain high GC contents as well (Higgs and Ran 2008, Satapathy et al. 2010).

The results of phenotypic and physiological characters of thermophilic bacterial isolates, MBT010 and MBT008 are in line with those reported by Bisht and Panda (2011), whereas the bacterial strain MBT011 is in agreement with outcomes of the studies by Shida et al. (1997) and for the thermophilic bacterial strains MBT006 and MBT018, our findings also correlate with the study of Mohammed in 2012, who isolated the three new species of moderately thermophilic bacteria from three hot springs, located in Jazan District, south-western of Saudi Arabia. They did the phylogenetic analysis of these strains using their 16S rDNA sequence and also studied the morphological, biochemical and physiological characteristics of the isolates (Mohammad A. Khiyami 2012). On the basis of phenotypical and physiological features, these isolated thermophiles also depicted consistent results with those of Rastogi et al. (2009).

In another study from Pakistan, Zahra et al in 2020 isolated strain AK9 from the hot water spring of Tattapani Azad Kashmir, Pakistan; extracted and purified cellulase enzyme which reserved its activity from 50-70°C and 3–7 pH. They reported that *B.amyloliquefaciens* AK9 can be used in bioconversion of lignocellulosic biomass to fermentable sugar (Amin et al. 2016a, Amin et al. 2016b,Zahra et al. 2020).

The phylogenetic analysis, based on 16S rRNA gene similarity, depicted that the strain MBT009 was affiliated with the species *Anoxybacillusgonensis* with 99.83% sequence similarity as shown in Fig. 3(A). Our results are consistent with the results of Dai et al. (2011) who had isolated the E13T strain from water-sediment samples from springs in Yunnan Province of China, closely related to the species of *A.flavithermus* (99.2% sequence similarity). These findings also resemble the study in which Chan et al. (2015) identified bacterial strains affiliated with genra *Anoxybacillus* and *Geobacillus* from a Malaysian hot spring. Their results revealed a clear domination of the genus *Anoxybacillus* represented by *A.thermarum*, *A.flavithermus* and *A.pushchinoensis* (Chan et al. 2015).

Our study correlates with the study of Balsam et al. (2017) in which they reported the morphological, microscopic, biochemical, molecular and physiological characterisation of ten isolates isolated from hot springs in Jordan; out of which nine isolates belonged to the genus *Bacillus*.

Anoxybacillus a relatively new genus, is alkali tolerant, Gram-positive rod, tested positive for catalase and oxidase activity. In Pakistan, this genus has been identified and characterised by Jabeen et al. in 2019 from the hot water spring in Chakwal (Jabeen et al. 2019).

In our study, one of the isolates namely MBT009 had nearest similarity (99.83%) with *Anoxybacillusgonensis* which was related to the study of Belduz et al. (2003), who had isolated the thermophilic strain G2T (which showed strong homology > 97% with *A.gonensis*) from mud and water samples from the Gonen and Diyadin hot springs, respectively, located in the Turkish provinces of Balikesir and Agri. They had screened the strains for thermostable enzyme production and found that both strains had the ability to produce industrially-important enzymes (Belduz et al. 2003).

## Conclusion

Thermophiles are unique organisms with tremendous diversity and biological significance. Azad Jammu Kashmir is an unexplored area with blessed sources of hot springs. The microbial diversity of hot springs from Kotli AJK has been explored for the first time on the basis of morphological, biochemical and molecular markers. In this study, ten (10) strains of thermophilic bacteria were isolated from different locations of geothermal hot springs of Tatapani. The 16s gene sequence revealed striking genetic variability in these isolates. These strains were related to genus *Anoxybacillus* with significant levels of similarity. It was clearly demonstrated that the thermal water of Tatapani (Kotli) hot spring can be an important source of diversity of thermophilic bacteria which is of great significance for biotechnological processes at elevated temperatures. It is important to report that this is the first study to reveal this hot spring from a single common source for a diversity of numerous thermophillic bacteria, so their applications in the field of biotechnology can be exploited for the production of thermostable enzymes, as well as their metabolites can be used in various biotechnological processes. The study provided an authentic base for studies on complete genomic characterisation and are highly recommended for generating more deatiled information of novel isolates.

## Figures and Tables

**Table 1.** Total cell count of live and cultivable cells in samples.

**Sample**	**Temperature**	**pH**	**CFU/ml**
**MBT006**	67	7	6.0×10^3^
**MBT007**	52	6.9	5.2×10^3^
**MBT008**	49	7	4.7×10^3^
**MBT009**	52	7.1	5.1×10^3^
**MBT010**	51	6.9	Not Determined
**MBT011**	53	7	2.9×10^3^
**MBT012**	54	6	2.1×10^3^
**MBT013**	52	6	1.4×10^4^
**MBT014**	51	7	5.7×10^3^
**MBT018**	49	7	5.2×10^3^

**Table 2.** Morphological characteristics.

**Sample**	**Shape**	**Margin**	**Visual colour**	**Colour under microscope**	**Elevation**	**Texture**
MBT006	Regular	Smooth	White-creamy	Dark brown	Raised	Creamy
MBT007	Irregular	Smooth	Grey-yellow	Dark brown	Raised	Creamy
MBT008	Regular	Smooth	white	Golden	Flat	Creamy
MBT009	Irregular	Rough	White-creamy	Dark brown	Raised	Sticky
MBT010	Irregular	Smooth	Grey-brown	Dark brown	Raised	Creamy
MBT011	Regular	Rough	White	Brown	Raised	Creamy
MBT012	Irregular	Smooth	Off-white	Dark brown	Flat	Creamy
MBT013	Regular	Smooth	Yellow	Pale	Raised	Sticky
MBT014	Regular	Rough	Pale	Brown	Raised	Creamy
MBT018	Irregular	Smooth	Off-white	Golden	Raised	Creamy

**Table 3.** Cell morphology of bacterial isolates.

**Strains**	**Cell shape**	**Gram staining**	**Arrangement**
**MBT006**	Rod	+ve	Pairs/clusters
**MBT007**	Rod	+ve	Pairs
**MBT008**	Rod	+ve	Pairs
**MBT009**	Rod	+ve	Clusters
**MBT010**	Rod	+ve	Pairs/clusters
**MBT011**	Rod	+ve	Pairs/clusters
**MBT012**	Rod	+ve	Pairs/clusters
**MBT013**	Rod	+ve	Pairs
**MBT014**	Rod	+ve	Pairs/clusters
**MBT018**	Rod	+ve	Clusters

**Table 4.** Biochemical characteristics of bacterial isolates.

**Tests**	**MBT006**	**MBT007**	**MBT008**	**MBT009**	**MBT010**	**MBT011**	**MBT012**	**MBT013**	**MBT014**	**MBT018**
**β-galactosidase**	+	­+	+	+	+	+	+	+	+	+
**L-arginine**	_	+	_	_	+	+	_	+	+	+
**L-lysine**	_	_	_	+	+	_	_	_	_	_
**Citrate utilisation**	+	+	+	+	+	+	+	+	+	+
**H_2_S production**	_	+	+	+	_	_	+	+	_	_
**L-tryptophane**	+	+	_	+	+	+	+	_	+	_
**Indole production**	_	_	_	_	_	+	+	_	_	_
**Acetoin production**	+	+	+	_	+	+	+	_	+	+
**Gelatinase**	_	+	+	+	+	+	_	+	+	+
**D-glucose**	_	_	+	+	_	+	+	+	_	_
**D-manitol**	_	_	_	_	_	_	_	_	_	_
**Inositol**	­_	_	_	_	_	_	_	_	_	_
**D-sorbitol**	­_	_	_	_	_	_	_	_	_	_
**L-rhamnose**	­+	_	_	_	+	_	_	_	_	_
**D-sucrose**	­_	_	_	_	_	_	_	_	_	_
**D-melibiose**	­+	_	_	+	+	_	_	_	+	_
**Amygdalin**	­+	_	+	+	_	_	_	_	_	_
**L-arabinose**	­+	+	+	_	_	+	_	_	_	_
**Cytochrome-oxidase**	+	+	+	_	+	_	+	+	+	+

**Table 5.** Antibiotic resistance spectra of thermophilic bacterial strains by the disc diffusion method.

**Strains**	**Ax**	**MET**	**LEV**	**SXT**	**Cip**	**CFM**	**PRL**	**AZM**	**CLR**	**LNZ**
**MBT006**	24 (S)	**0** (R)	32 (S)	25 (S)	30 (S)	16 (S)	18 (S)	0 (R)	41 (S)	17 (S)
**MBT007**	29 (S)	0 (R)	25 (S)	16 (S)	42 (S)	0 (R)	34 (S)	21 (S)	0 (R)	20 (S)
**MBT008**	17 (S)	0 (R)	24 (S)	0 (R)	16 (S)	24 (S)	19 (S)	0 (R)	30 (S)	0 (R)
**MBT009**	26 (S)	0 (R)	24 (S)	19 (S)	0 (R)	17 (S)	0 (R)	23 (S)	0 (R)	0 (R)
**MBT010**	16 (S)	0 (R)	22 (S)	24 (S)	41 (S)	0 (R)	18 (S)	0 (R)	26 (S)	16 (S)
**MBT011**	30 (S)	0 (R)	20 (S)	26 (S)	30 (S)	19 (S)	16 (S)	0 (R)	32 (S)	26 (S)
**MBT012**	21 (S)	16 (S)	24 (S)	0 (R)	17 (S)	30 (S)	36 (S)	18 (S)	0 (R)	0 (R)
**MBT013**	24 (S)	0 (R)	32 (S)	20 (S)	0 (S)	30 (S)	18 (S)	0 (R)	26 (S)	32 (S)
**MBT014**	17 (S)	0 (R)	30 (S)	16 (S)	28 (S)	18 (S)	26 (S)	0 (R)	32 (S)	30 (S)
**MBT018**	18 (S)	0 (R)	18 (S)	34 (S)	26 (S)	18 (S)	30 (S)	17 (S)	16 (S)	20 (S)

**Table 6.** List of thermophillic bacteria identified on the basis of 16S rRNA genes.

**Isolate code**	**Closest affiliation**	**Accession number**	**Percentage similarity**
**MBT008**	*Anoxybacilluskamchatkensis*	OM918284	100
**MBT009**	*Anoxybacillusgonensis*	OM918285	99.83
**MBT012**	*Anoxybacillusmongoliensis*	OM918286	99.57
**MBT014**	*Anoxybacillustengchongensis*	OM918287	99.43
**MBT018**	*Anoxybacillusskarvacharensis*	OM918288	98.70
